# Risk factors for venous port migration in a single institute in Taiwan

**DOI:** 10.1186/1477-7819-12-15

**Published:** 2014-01-14

**Authors:** Wen-Chieh Fan, Cheng-Han Wu, Ming-Ju Tsai, Ying-Ming Tsai, Hsu-Liang Chang, Jen-Yu Hung, Pei-Huan Chen, Chih-Jen Yang

**Affiliations:** 1 Department of Surgery, Kaohsiung Medical University, 68 ChungHwa 3rd Road, Cianjin District, Kaohsiung City 80145, Taiwan; 2 Department of Internal Medicine, Kaohsiung Municipal Ta-Tung Hospital, Kaohsiung Medical University Hospital, Kaohsiung Medical University, 68 ChungHwa 3rd Road, Cianjin District, Kaohsiung City 80145, Taiwan; 3 Division of Pulmonary and Critical Care Medicine, Department of Internal Medicine, Kaohsiung Medical University Hospital, Kaohsiung, Taiwan; 4 School of Medicine, College of Medicine, Kaohsiung Medical University, Kaohsiung, Taiwan

**Keywords:** Venous port, Migration

## Abstract

**Background:**

An implantable port device provides an easily accessible central route for long-term chemotherapy. Venous catheter migration is one of the rare complications of venous port implantation. It can lead to side effects such as pain in the neck, shoulder, or ear, venous thrombosis, and even life-threatening neurologic problems. To date, there are few published studies that discuss such complications.

**Methods:**

This retrospective study of venous port implantation in a single center, a Taiwan hospital, was conducted from January 2011 to March 2013. Venous port migration was recorded along with demographic and characteristics of the patients.

**Results:**

Of 298 patients with an implantable import device, venous port migration had occurred in seven, an incidence rate of 2.3%. All seven were male and had received the Bard port Fr 6.6 which had smaller size than TYCO port Fr 7.5 and is made of silicon. Significantly, migration occurred in male patients (*P* = 0.0006) and in those with lung cancer (*P* = 0.004). Multivariable logistic regression analysis revealed that lung cancer was a significant risk factor for port migration (odds ratio: 11.59; *P* = 0.0059). The migration rate of the Bard port Fr 6.6 was 6.7%. The median time between initial venous port implantation and port migration was 35.4 days (range, 7 to 135 days) and 71.4% (5/7) of patients had port migration within 30 days after initial port implantation.

**Conclusions:**

Male sex and lung cancer are risk factors for venous port migration. The type of venous port is also an important risk factor.

## Background

Venous port implantation is widely used for the safe delivery of systemic chemotherapy in patients with cancer. However, various complications have been documented and the total complication rate ranges from 0.4% to 29% [1-6]. Catheter migration is a rare complication with an unknown cause that occurs in about 0.9 to 2% of patients [3,5,7,8]. It can lead to side effects such as pain in the neck, shoulder, or ear, venous thrombosis [9-12], and even life-threatening neurologic problems [13-15]. Because of its rarity, very few studies have been published that extensively tackled venous catheter migration [3,7]. This retrospective study is an investigation of venous port migration in a hospital in Taiwan. Related literature is also reviewed.

## Methods

All patients who underwent venous port implantation (BardPort® 6.6 Fr implantable port, NJ, USA (Bard port) or Autosuture Chemosite® Fr 7.5, Tyco healthcare group, Connecticut, USA (TYCO port)) for chemotherapy at Kaohsiung Municipal Ta-Tung Hospital between 1 January 2011 and 31 March 2013, were retrospectively evaluated. The Bard port® was made of silicon while the Autosuture Chemosite® was made of polyurethane (PU). The procedures were all performed by an experienced surgeon (Dr. WC Fan) under local anesthesia. The vessel cut-down method was used for catheter cannulation. After venostomy, the distal end of the entry vessel was controlled and the catheter was inserted via the superior vena cava. The cephalic vein was the first choice for entry exploration and occasionally, the subclavian vein was the point of entry if the cephalic vein was difficult to access.

All locations of the implanted venous ports were confirmed by fluoroscopy and post-operative x-rays. The surgeon ensured that all of the tips of the venous catheters were located at the junction of the superior vena cava and right atrium (cavo-atrial junction) intra-operatively. The cavo-atrial junction was the point at which the superior vena cava met and joined the superior wall of the right atrium. For purposes of radiographic visualization, the most reliable indicators of the junction were the carina and the overlying vertebrae. The junction lay two vertebral body units below the carina [16].

**Figure 1:**
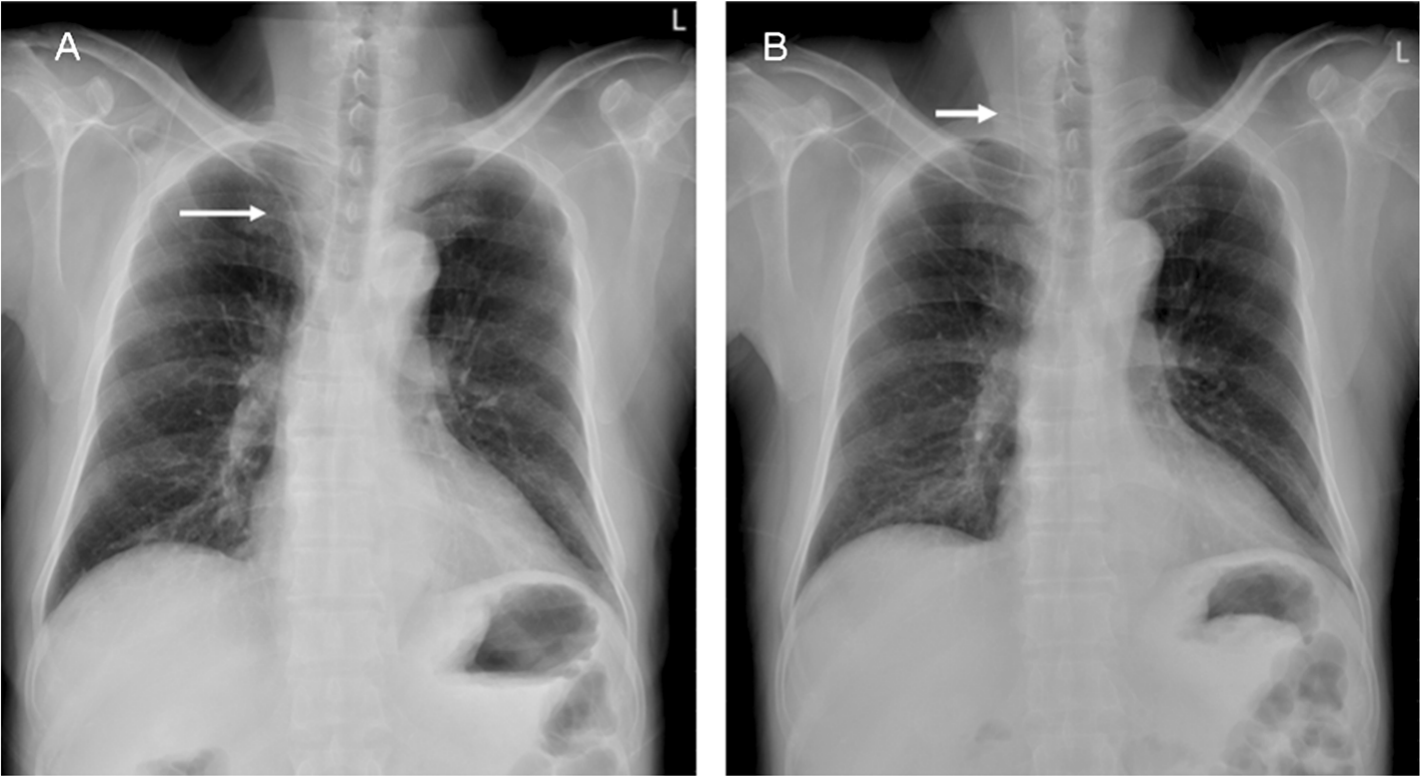
A 48-year-old man who underwent (A) venous port implantation experienced (B) catheter migration after 21 days.

The term ‘venous port migration’ referred to a venous port that was not in the correct position. This was diagnosed based on chest x-ray taken before the course of chemotherapy in a patient (Figure 1). The Institutional Review Board of Kaohsiung Medical University Hospital approved this study.

Data were entered and analyzed using the JMP statistical software (version 9.0, SAS Institute Inc., Cary, NC, USA). Demographic data, underlying cancer, and related covariates were compared between the group with and without port migration using the Fisher’s exact test for categorical variables or Wilcoxon rank sum test for continuous variables. To identify the major factors associated with port migration, multivariable logistic regression was performed. Statistical significance level was set at *P* < 0.05.

## Results

A total of 298 patients with mean (± standard deviation) age of 61.4 ± 12.3 years were enrolled. Among them, 145 (48.7%) were male (Table 1). Moreover, 104 patients received the Bard port and 194 received the TYCO port. The patients who received the Bard port implantation were older, with male predominance. More patients in the Bard port group received their port through the right side via the cephalic vein. In terms of port migration, the Bard port had a significantly higher migration rate, up to 6.7%, compared to 0% of the TYCO port (*P* = 0.0006).

**Table 1:** Baseline characteristics of the patients using different ports

	**All patients**		**Bard port**		**TYCO port**		***P*** **value**
	**n**	**%**	**n**	**%**	**n**	**%**	
Patient number	298		104		194		
Age							0.0116
mean ± standard deviation	61.4 ± 12.3		63.9 ± 11.3		60.1 ± 12.7		
median (inter-quartile range)	61 (54 to 70.8)		63.1 (56.3 to 72)		60 (51 to 69)		
Sex							0.0034
Female	153	51.3%	41	39.4%	112	57.7%	
Male	145	48.7%	63	60.6%	82	42.3%	
Body mass index							0.1077
mean ± standard deviation	24.2 ± 6.8		23.4 ± 3.6		24.7 ± 8.0		
medium (inter-quartile range)	23.6 (21.1 to 26.4)		23.4 (21.1 to 25.4)		23.9 (21.0 to 26.7)		
Migration							0.0006
No	291	97.7%	97	93.3%	194	100.0%	
Yes	7	2.3%	7	6.7%	0	0.0%	
Side							< 0.0001
Left	97	32.6%	19	18.3%	78	40.2%	
Right	201	67.5%	85	81.7%	116	59.8%	
Entering vessel							< 0.0001
Cephalic vein	261	87.6%	102	98.1%	159	82.0%	
Subclavian vein	37	12.4%	2	1.9%	35	18.0%	
Malignancy (indication for the port)							
Head and neck tumor	17	5.7%	4	3.9%	13	6.7%	
Lung cancer	59	19.8%	32	30.8%	27	13.9%	
Esophageal cancer	2	0.7%	0	0.0%	2	1.0%	
Breast cancer	55	18.5%	4	3.9%	51	26.3%	
Gastric cancer	14	4.7%	7	6.7%	7	3.6%	
Colorectal cancer	64	21.5%	29	27.9%	35	18.0%	
Hepatobiliary and pancreatic tumor	8	2.7%	3	2.9%	5	2.6%	
Ovary cancer and cervical cancer	26	8.7%	7	6.7%	19	9.8%	
Urological cancer	37	12.4%	12	11.5%	25	12.9%	
Leukemia and lymphoma	11	3.7%	3	2.9%	8	4.1%	
Other malignancy	3	1.0%	1	1.0%	2	1.0%	
Malnutrition, no malignancy	2	0.7%	2	1.9%	0	0.0%	
Lung cancer							0.0007
Yes	59	19.8%	32	30.8%	27	13.9%	
No	239	80.2%	72	69.2%	167	86.1%	

Based on the occurrence of port migration, the patients were further classified into the migration group and non-migration group (Table 2). The mean age and mean body mass index (BMI) did not differ significantly between the two groups. Venous port migration occurred in only seven patients in this retrospective study. All of them were male, used the Bard port, and received their port via the cephalic vein. Five (71.4%) of them were lung cancer patients.

**Table 2:** Characteristics of the patients with and without port migration

	**Migration group**		**Non-migration group**		***P*** **value**
	**n**	**%**	**n**	**%**	
Patient number	7		291		
Age					0.1181
mean ± SD	68.1 ± 10.7		61.2 ± 12.4		
median (inter-quartile range)	71 (62 to 74)		61 (54 to 70.3)		
Gender					0.0060
Female	0	0.0%	153	52.6%	
Male	7	100.0%	138	47.4%	
Body mass index					0.3211
mean ± standard deviation	24.9 ± 1.8		24.2 ± 6.9		
medium (inter-quartile range)	24.7 (23.3 to 26.9)		23.6 (21 to 26.4)		
Venous port type					0.0006
Bard Fr 6.6	7	100.0%	97	33.3%	
TYCO Fr 7.5	0	0.0%	194	66.7%	
Side					0.4338
Left	1	14.3%	96	33.0%	
Right	6	85.7%	195	67.0%	
Entering vessel					0.6025
Cephalic vein	7	100.0%	254	87.3%	
Subclavian vein	0	0.0%	37	12.7%	
Malignancy (indication for the port)					
Head and neck tumor	0	0.0%	17	5.8%	
Lung cancer	5	71.4%	54	18.6%	
Esophageal cancer	0	0.0%	2	0.7%	
Breast cancer	0	0.0%	55	18.9%	
Gastric cancer	1	14.3%	13	4.5%	
Colorectal cancer	0	0.0%	64	22.0%	
Hepatobiliary and pancreatic tumor	0	0.0%	8	2.7%	
Ovary cancer and cervical cancer	0	0.0%	26	8.9%	
Urological cancer	0	0.0%	37	12.7%	
Leukemia and lymphoma	1	14.3%	10	3.4%	
Other malignancy	0	0.0%	3	1.0%	
Malnutrition, no malignancy	0	0.0%	2	0.7%	
Lung cancer					0.0040
Yes	5	71.4%	54	18.6%	
No	2	28.6%	237	81.4%	

Multivariable logistic regression analysis of the factors related to port migration revealed that lung cancer was a significant risk factor for port migration (odds ratio (OR): 11.59, 95% confidence interval (CI): 2.25 to 87.73; *P* = 0.0059). Sex and venous port type were not included in the model because all of the patients in the migration group were male and had been fitted with the Bard port (Table 3).

**Table 3:** Multivariable logistic regression analysis of factors affecting port migration

**Variables**	**Odds Ratio**	**95%CI**	***P*** **value**
All study subjects			
Age > 60 versus age ≤ 60	4.85	(0.77 to 94.61)	0.1542
BMI ≥ 24 versus BMI < 24	2.60	(0.50 to 15.34)	0.2578
Right versus left	2.15	(0.31 to 42.73)	0.4980
Lung cancer versus others	11.59	(2.25 to 87.73)	0.0059
Patients using Bard port			
Age > 60 versus age ≤ 60	4.28	(0.64 to 86.22)	0.2019
BMI ≥ 24 versus BMI < 24	2.46	(0.47 to 14.54)	0.2877
Right versus left	1.22	(0.16 to 25.72)	0.8638
Lung cancer versus others	7.59	(1.47 to 58.03)	0.0235
Male patients			
Age > 60 versus age ≤ 60	3.35	(0.51 to 68.47)	0.2826
BMI ≥ 24 versus BMI < 24	2.45	(0.47 to 14.56)	0.2911
Right versus left	2.27	(0.33 to 45.38)	0.4704
Lung cancer versus others	7.03	(1.37 to 53.15)	0.0283
Lung cancer patients			
Age > 60 versus age ≤ 60	2.59	(0.34 to 53.55)	0.4152
BMI ≥ 24 versus BMI < 24	1.65	(0.19 to 12.53)	0.6216
Right versus left	1.32	(0.14 to 28.65)	0.8201

The median time between initial venous port implantation and port migration was 35.4 days (range, 7 to 135 days), 71.4% of which occurred within 30 days after initial port implantation.

There were no complications associated with the port migration as it was promptly detected before intravenous chemotherapy was administered. All of the patients successfully underwent surgical revision of the venous port and no recurrence of port migration was noted till the end of the study.

## Discussion

This report demonstrates that the type of venous port may lead to different migration rates. Moreover, male sex and lung cancer patients also have significantly higher migration rates.

An implantable port device provides easy access for long-term chemotherapy. A recent retrospective analysis of more than 3,000 chest-port placements by interventional radiologists provides a good analysis of outcomes, including a nearly 100% technical success rate and an overall complication rate of 11.8% (1.3% peri-procedural, 3.3% early, and 9.4% late complications) [17]. Early complications include pneumothorax, hematoma, malposition, embolism, or arrhythmia, which are often related to the placement technique. Delayed complications include skin necrosis, infection, catheter fracture, occlusion, thrombosis, and migration [1,2,6-8,17].

Infectious and thrombotic issues dominate port complications. The reported rates of long-term venous access infections range from 0.6% to 27% and depend on catheter location, catheter type, and patient’s immune status [18]. Spontaneous venous port migration is rare and occurs in about 0.9 to 2% of patients, according to published articles [3,5,7,8]. In one study, the indicated incidence of catheter migration is about 0.04/1,000 catheter days.

In fluoroscopy-assisted port catheter implantation, primary migration (intra-operative) of the catheter tip is rare but secondary migration (post-operative) may occur due to high intra-thoracic pressure, arm movement, or other unknown causes. This current study reveals a similar incidence of venous port migration of 2.34%. However, there is a significantly higher incidence of venous port migration in patients whose venous port is Bard Fr 6.6 compared to the Autosuture Chemosite® Fr 7.5. Some reasons can be posited. The smaller caliber port (Bardport® Fr 6.6) may be more flexible and have more potential of migrating to the ipsilateral internal jugular vein. Furthermore, BardPort® is made of silicon whereas the Autosuture Chemosite® is made of PU. Silicon is more flexible than PU and this may account for the higher migration rate.

In a report of Wu *et al*., there is no difference between any port types. However, the incidence of migration in the Bard Fr 6.6 is as high as 3.69% (15/406), much higher than that of other types [3].

The most common migration site is the internal jugular vein. Sometimes, catheters migrate to the peri-cardiophrenic vein and cause cardiac tamponade [19]. Cough-induced port migration to the right axillary vein has also been reported. Other migration/malposition sites are the left subclavian vein, right internal thoracic vein, inferior thyroid vein, left brachial vein, and right brachiocephalic vein. All of the seven cases involve catheter migration into the ipsilateral internal jugular vein. In addition, a high infusion flow rate can also make the tip migrate.

Wu *et al*. have stated that shallow catheter-tip location and the presence of lung cancer are risk factors for catheter migration. Strategies that ensure low catheter-tip location and avoid increased thoracic pressure may be useful preventive measures [3]. Once migration is detected, prompt correction is important. A catheter tip position in the right atrium or ventricle may cause cardiac arrhythmia, perforation, tamponade, or thrombosis. When the central venous catheter tip is located in an undesirable site, the injected drugs or fluid will also directly enter a small caliber vein and subsequently induce complications like neck pain, shoulder pain, ear pain, venous phlebitis, or thrombosis. Some venous port migrations have been detected following chest x-ray in patients who complain of right neck pain.

The inadvertent infusion of irritant drugs may be life-threatening if neurologic complications and cortical vein thrombosis occur [13,14]. Fortunately, no complications developed in the study period because each migration event was promptly detected prior to chemotherapy.

The mechanism of venous port migration remains unclear but physical forces acting on the catheter has been proposed, including increased intra-thoracic pressure due to coughing, sneezing, or weight lifting, changes in body position, or physical movements like abduction or adduction of the arms or hyper-extension of the shoulder [3,10]. Among these possible mechanisms, cough is the most common in several case reports [12]; severe cough can generate up to 300 mmHg of intra-thoracic pressure against a closed glottis, followed by forceful expulsion of air and secretions via the glottic opening. Vigorous changes in intra-thoracic pressure may result in herniation of a short segment of the shaft of the catheter into the jugular vein [20]. Repeated cough may cause progressive herniation and eventually complete the caudal migration of the catheter tip [3]. Moreover, cough is the most common symptom in patients with lung cancer. This hypothesis may explain why, in the study by Wu *et al*. and in the present study, lung cancer patients have a higher incidence of migration than other cancers. A high infusion flow rate can also lead to tip migrate, as reported.

In the current study, venous port migrations occur early after implantation. In a swine model, central venous catheters have a partial or circumferential mixed cellular and non-cellular covering consisting of smooth muscle cells, thrombus, and areas with endothelial cell populations. Less prominent cellularity and more prominent collagen content develop after 30 to 45 days. With longer catheter in-dwelling time, an endothelial layer, indistinguishable from the adjacent vein wall, covers the catheter surface [21]. The experiment may explain why migration occurs within 30 days in most of the patients in the present study.

Once migration is detected, prompt revision is important to avoid complications. Re-positioning through either surgery-based revision or radiologic interventional procedure shows very high initial success rates [7,12,22]. The trans-femoral snaring technique is also a quick and easy method to re-position the catheter tip [23].

A limitation of this study is its small case number; therefore further large-scale studies are warranted.

## Conclusion

This is the first study to report that the type of venous port may affect the port migration rate. Male sex and lung cancer patients also have significantly higher migration rates. Port migration often occurs within 30 days after the initial implantation. Periodic check-ups by chest X-ray of catheter location are crucial for detecting catheter tip migration and for early intervention to prevent potential complications.

## Abbreviations

BMI: Body mass index.

## Competing interests

The authors declare that they have no competing interests.

## Authors’ contributions

CJY designed the study. WCF and CHW drafted the manuscript. MJT did the analysis. YMT and HLC participated in study design and data collection. JYH and PHC collected data and processed the data. All of the authors read and approved the final manuscript.
